# Nanomedicine targets iron metabolism for cancer therapy

**DOI:** 10.1111/cas.15250

**Published:** 2022-02-07

**Authors:** Liangru Lin, Hanqing Chen, Ruifang Zhao, Motao Zhu, Guangjun Nie

**Affiliations:** ^1^ College of Pharmaceutical Science Jilin University Changchun China; ^2^ Department of Gastroenterology Guangzhou Digestive Disease Center Guangzhou First People’s Hospital School of Medicine South China University of Technology Guangzhou China; ^3^ CAS Key Laboratory for Biomedical Effects of Nanomaterials and Nanosafety CAS Center of Excellence in Nanoscience National Center for Nanoscience and Technology Beijing China

**Keywords:** cancer therapy, ferroptosis, iron metabolism, iron reductive therapy, nanodrug delivery system

## Abstract

Iron is an essential element for cell proliferation and homeostasis by engaging in cell metabolism including DNA synthesis, cell cycle, and redox cycling; however, iron overload could contribute to tumor initiation, proliferation, metastasis, and angiogenesis. Therefore, manipulating iron metabolisms, such as using iron chelators, transferrin receptor 1 (TFR1) Abs, and cytotoxic ligands conjugated to transferrin, has become a considerable strategy for cancer therapy. However, there remain major limitations for potential translation to the clinic based on the regulation of iron metabolism in cancer treatment. Nanotechnology has made great advances for cancer treatment by improving the therapeutic potential and lowering the side‐effects of the proved drugs and those under various stages of development. Early studies that combined nanotechnology with therapeutic means for the regulation of iron metabolism have shown certain promise for developing specific treatment options based on the intervention of cancer iron acquisition, transportation, and utilization. In this review, we summarize the current understanding of iron metabolism involved in cancer and review the recent advances in iron‐regulatory nanotherapeutics for improved cancer therapy. We also envision the future development of nanotherapeutics for improved treatment for certain types of cancers.

## Graphical abstract

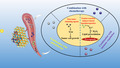

## 1 INTRODUCTION

Iron is an essential element for cell survival, and functions in proteins mainly containing heme and iron‐sulfur clusters, such as metalloproteins and enzymes that are involved in many vital metabolic processes and energy homeostasis including mitochondrial respiration, oxygen sensing and transport, citric acid cycle, and DNA biosynthesis.^1^ In mammalian cells, iron exists mostly in the form of heme, which is a subunit of hemoglobin, in the ferrous state (Fe^2+^), whereas excessive free iron is considered cytotoxic through catalyzing Fenton’s reaction and generates reactive oxygen species (ROS) such as hydroxyl radical.^2^ Reactive oxygen species can induce oxidative damage by attacking cellular membranes, proteins, and DNA. Some evidence has shown the correlation of dysregulation of iron with the development of cancer in humans. Cancer cells reshape the pathways of iron metabolism to meet the high iron demand during tumor growth and malignancy.^3^ The vigorous aerobic glycolysis process of tumor cells is closely related to iron metabolism. However, it is still debatable whether iron dysregulation is the cause or the consequence of tumor development, although iron metabolism is well recognized to be perturbed in cancer cells.^4^, ^5^ Iron also plays a vital role in tumor progression and metastasis due to its role in tumor cell survival and tumor microenvironment reprogramming.^6^ Thus, iron metabolism‐targeted therapy, such as iron depletion, has been explored as an effective strategy for intervening in oncogenesis and tumor metastasis.^7^, ^8^

Iron chelators have been widely used to treat iron overload diseases and have attracted increasing attention for cancer therapy. Previous studies have reported that iron chelators, such as deferoxamine (DFO), deferiprone, ciclopirox, or deferasirox, could significantly inhibit cancer cell proliferation by inducing cell cycle arrest and apoptosis.^9^ Although iron chelators have shown great potential in preclinical cancer models, small‐molecule iron chelators could cause adverse side‐effects, such as infection, gastrointestinal bleeding, renal failure, and liver fibrosis.^10^ In addition, the lack of tumor cell specificity and poor pharmacokinetics also limit their therapeutic potential and further clinical application.^11^ Therefore, overcoming these challenges is demanded to improve the therapeutical efficacy of iron chelators for tumor treatment.

In addition to iron depletion therapy mediated by iron chelators, ferroptosis has attracted considerable interest due to its involvement in immunity, development, and various pathological scenarios as a novel iron‐dependent nonapoptotic cell death.^12^ The activity of ferroptosis is mainly associated with the bioavailable Fe^2+^. Emerging evidence indicates that the iron‐mediated Fenton reaction plays an important role in ferroptosis induction. Fe^2+^ is reduced from imported ferric iron (Fe^3+^) by duodenal cytochrome B and transported by the divalent metal transporter 1 in the endosome. Free Fe^2+^ might participate in the Fenton reaction together with hydrogen peroxide (H_2_O_2_), resulting in the production of lethal ROS and regeneration of Fe^3+^. Therefore, the investigation of ferroptosis that specifically targets cancer cells highlights the promising role of ferroptosis induction in cancer treatment. Ferroptosis‐based cancer therapy has been reported to bypass the drawbacks of well‐established cancer therapeutics (eg, chemotherapy or radiotherapy), as well as emerging cancer immunotherapy. Therefore, targeting iron metabolism and inducing ferroptosis have both been regarded as promising strategies for cancer therapy. However, some ferroptosis inducers, such as ferroptosis‐suppressing cystine/glutamate antiporter system inhibitors, hardly reach sufficient concentrations in targeted tumor tissues, resulting in minimal therapeutic effect.^13^ Additionally, it is possible that systemic inhibition of iron metabolism would generate off‐target effects, which leads to undesirable toxicities.

Due to its multifunctional capacity and diverse biological activities, nanoenabled drug encapsulation and delivery technology have been explored to provide an innovative therapeutic regime in the treatment of cancer.^14^, ^15^, ^16^, ^17^ By increasing efficacy and reducing adverse side‐effects of cytotoxic drugs, some liposomes and albumin‐based therapies have been approved by the US FDA and successfully improved the clinical performance of chemotherapeutics in cancer treatment, such as Doxorubicin hydrochloride and Paclitaxel.^18^ With the growing understanding of human physiology and pathology, many multifunctional nanomaterials have been designed and synthesized for the delivery of different types of drugs, including nucleic acid drugs, to enhance tissue and cell targeting and improve drug stability, pharmacokinetics, and biodistribution in cancer therapy. In addition, other novel therapeutics, for example, chemodynamic therapy, can induce tumor cell death and encourages attention for tumor‐specific therapy through decomposing intertumoral ROS that rely on iron‐mediated Fenton or Fenton‐like reactions, which utilize Fe^2+^ and Fe^3+^ in this process.^19^ Therefore, iron metabolism‐based nanomedicine and the corresponding combination therapy could provide a novel paradigm for cancer treatment. In this review, we summarize the recent progress in iron metabolism‐based cancer therapeutic strategies with a focus on iron chelator‐based nanostructures and ferroptosis‐inducing nanotherapeutics (Table 1).

**TABLE 1:** Nanotherapeutics for cancer treatment through iron‐related mechanisms

Strategies	Name	Nanoparticle type	Bioactive compound	Auxiliary method	Indication(s)	Status
Treatment of tumors by iron removal	TNP‐DFO‐YC1	Liposomes	DFO	HIF1α inhibitor	Pancreatic cancer	In vivo^41^
	Dp44mT‐NPs	Polymeric PLGA	Dp44mT	–	Malignant glioma	In vitro^37^
Nanotechnology‐enabled induction of ferroptosis	Feraheme	Iron oxide NPs	Fe_3_O_4_	–	Leukemia, early mammary cancers, lung cancer metastases in liver and lungs	In vivo^48^, ^49^
	LPO generator	Liposomes	FAC	–	Mammary cancer	In vivo^54^
	GFD NCs	DMSN	Fe_3_O_4_	GOD	Mammary cancer, malignant glioma	In vivo^55^
	SnFe_2_O_4_ Ncs	Nanocrystals	SnFe_2_O_4_	–	Colon cancer	In vitro^56^
	Nanolongan	UCNP	Fe^3+^	DOX	Mammary cancer	In vivo^57^
	ACC@DOX. Fe^2+^‐CaSi‐PAMAM‐FA/mPEG	ACC	Fe^2+^	DOX	Mammary cancer, melanoma	In vivo^58^
	SPFeN	SP_C_	Fe^3+^	PTT	Mammary cancer	In vivo^59^
	HSN	pTBCB‐PEG	Fe^2+^	PTT	Mammary cancer and metastases in liver and lungs	In vivo^60^
	TTIS	Graphdiyne oxide	Fe_3_O_4_	PTT	Mammary cancer	In vivo^61^
	Pa‐M/Ti‐NCs	Leukocyte membrane‐coated NCs	Fe_3_O_4_	Ti/Pa	Mammary tumor, melanoma	In vivo^64^

Note: –, to separate the essential components within the same nanostructure; ACC, amorphous calcium carbonate; CaSi, silica‐calcium carbonate; DFO, deferoxamine; DMSN, dendritic mesoporous silica nanoparticle; DOX, doxorubicin; Dp44mT, di‐2‐pyridyl ketone‐4,4‐dimethyl‐3‐thiosemicarbazone; FA, folic acid; FAC, ferric ammonium citrate; GFD, GOD‐Fe_3_O_4_@DMSN; GOD, glucose oxidase; HIF1α, hypoxia inducible factor 1 subunit α; HSN, hybrid semiconductor nanoenzyme; LPO, lipid peroxide; M;, membrane; NC, nanocatalyst; Ncs, nanocrystals; NP, nanoparticle; Pa, programmed cell death‐1 Ab; PAMAM, polyamidoamine; PLGA, poly (lactic‐co‐glycolic acid); pTBCB, PEGylated poly[(thiadiazoloquinoxaline‐alt‐benzodithiophene)‐ran‐(cyclopentadithiophene‐alt‐benzodithiophene)]; PTT, photothermal therapy; SPc, semiconducting polycomplex; SPFeN, iron‐chelated semiconductor multicomposite nanoparticle; Ti‐NC, transforming growth factor‐β inhibitor; TTIS, tumor‐targeted sponge iron; UCNP, up‐conversion nanoparticles; YC1, HIF1α inhibitor lificiguat.

## 2 SUMMARY OF THE MOLECULAR MECHANISM OF IRON PERTURBATION IN TUMORIGENESIS AND TUMOR PROLIFERATION

Recent studies have indicated that iron overload is associated with the pathogenesis and progression of many human diseases, including cancer.^20^, ^21^, ^22^, ^23^ Increasing evidence reveals that iron overload contributes to tumor initiation and is associated with an increased risk of tumor metastasis.^24^, ^25^, ^26^ The accumulation of iron, as well as active oxygen/nitrogen and aldehydes catalyzed by iron, leads to DNA chain scission for tumorigenesis.^27^ Patients with serous epithelial ovarian cancer have shown increased hemosiderin in the fallopian tubes along with elevated iron overload‐mediated oxidative stress and frequent DNA mutations.^28^, ^29^ Persistent iron overload also inhibits the activity of a classical tumor suppressor, p53 protein, to promote oncogenesis and tumor metastasis.^30^ Iron also regulates key signaling pathways in tumors, including hypoxia‐inducible factor (HIF) and Wnt signaling pathways that promote tumor survival, progression, and invasion.

Compared with normal cells, rapid tumor cell growth is largely dependent on iron. The rates of iron uptake and iron consumption are both accelerated simultaneously, leading to a high metabolic level of iron.^31^ The disorder of iron metabolism in cancer cells is related to the upregulation of key genes responsible for cellular iron absorption and the downregulation of genes related to iron efflux.^4^ For example, hepcidin, ferritin, transferrin, and transferrin receptor 1 (TFR1) levels are higher in tumor cells than in normal cells. Transferrin receptor 1 is a transmembrane glycoprotein that can mediate the endocytosis of iron‐bound transferrin in mammalian cells for iron uptake. It is overexpressed in various cancers to meet the high iron demand of rapidly proliferating cells. By contrast, the iron transporter ferroportin, serving as a pump for iron export by binding to hepcidin, is expressed at a lower level in tumor cells than in normal cells. The iron transporter‐hepcidin regulatory axis is essential in the regulation of abnormal iron metabolism in tumor cells. Iron response protein regulates cell iron metabolism by modulating the expression of ferritin and TFR1 at the posttranscriptional level. The upregulation of ferritin promotes cancer proliferation and metastasis by increasing the accumulation of mitochondrial iron‐sulfur proteins.^32^, ^33^ Lipocalin‐2 is a protein involved in the bypass pathway of iron uptake, and its expression level increases in tumor tissues.^31^, ^34^, ^35^ Mitochondrial iron metabolism disorders can inhibit endogenous cell apoptosis. Mitochondrial ferritin is preferentially expressed in cells with high metabolic activity and oxygen consumption, which is consistent with its role in chelating iron and preventing oxygen‐derived redox damage. It can alleviate the cell death caused by hypoxia by isolating unreacted iron. Therefore, increasing mitochondrial ferritin expression in cells under hypoxia can prevent the tissue from damage caused by insufficient oxygen.^36^

## 3 IRON‐BASED NANOTHERAPEUTICS FOR CANCER THERAPY

### 3.1 Iron nanochelating agents

**FIGURE 1:**
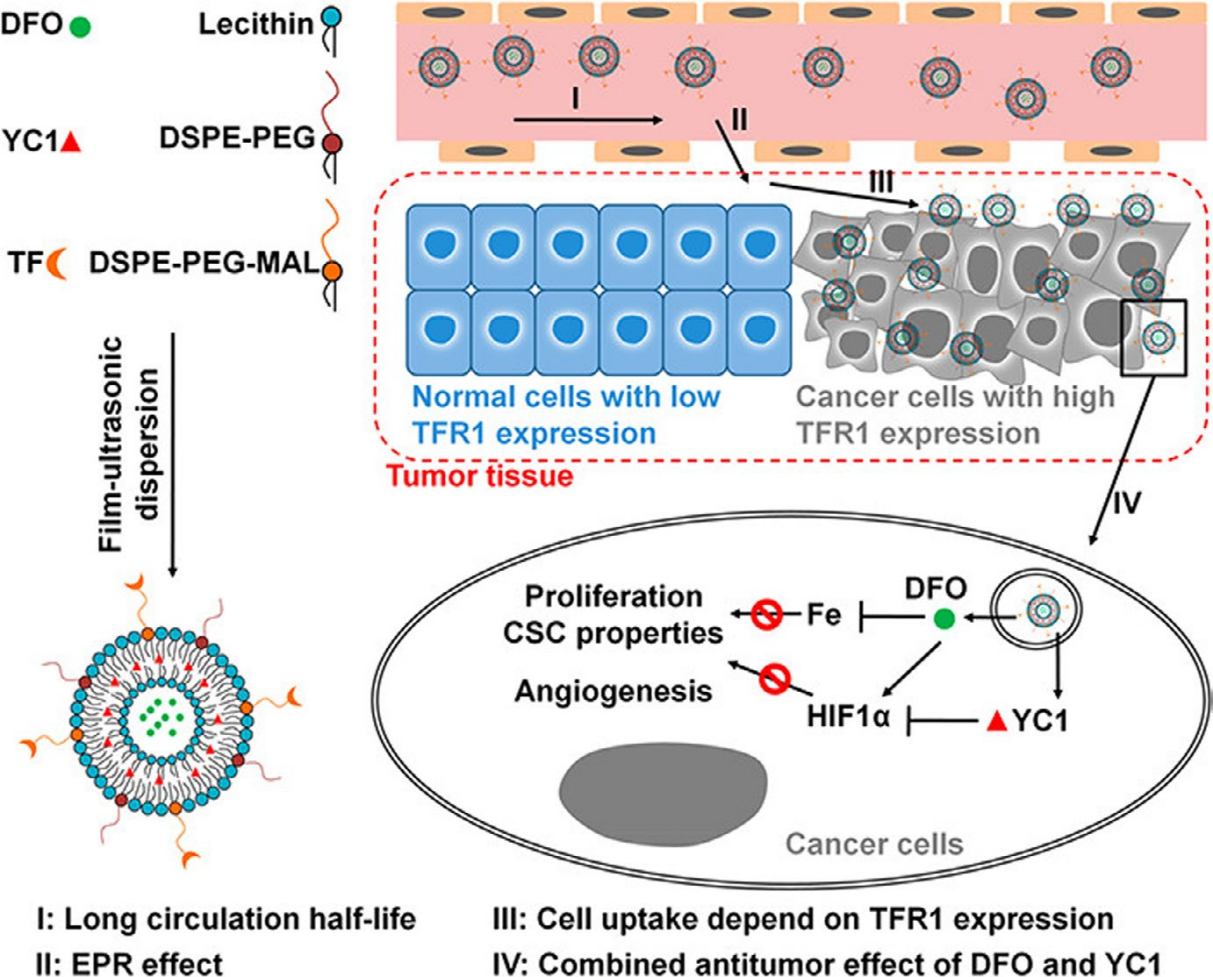
Assembly of deferoxamine (DFO)/lificiguat (YC1)‐loaded liposomes and the antitumor mechanism. DFO and YC1 were encapsulated into the hydrophilic and hydrophobic layers, respectively. The surface of the liposomes was decorated with transferrin by chemical cross‐linking. Nanoparticle‐encapsulated DFOs (TNP‐DFO‐YC1) show a much longer circulation half‐life than free DFO (I) and accumulate in tumor tissue through the enhanced permeability and retention (EPR) effect (II). TNP‐DFO‐YC1 was then selectively taken up by cancer cells that express high levels of transferrin receptor 1 (TFR1) on their surface (III). After the internalization of TNP‐DFO‐YC1, the drugs are released inside the cell, where they exert their antitumor effects (IV). Reprinted from Lang et al (2019),^41^ with permission from the American Chemical Society. CSC, cancer stem cell; DSPE‐PEG, distearoylphosphatidylethanolamine‐polyethylene glycol; HIF1α, hypoxia‐inducible factor 1α; MAL, maleimide; TF, transferrin

Nanotechnology‐based iron chelators have been extensively studied in cancer therapy.^9^ Novel iron nanochelators based on di‐2‐pyridylketone 4,4‐dimethyl‐3‐thiosemicarbazone (Dp44mT) have shown promise for the treatment of highly aggressive malignant tumors, for example, brain gliomas.^37^ Deferoxamine, the most commonly used iron chelator approved by the FDA,^38^ has recently been proposed as a potential therapeutic drug for patients with neuroblastoma, leukemia, prostate cancer, and hepatocellular carcinoma through specifically chelating iron in tumor cells.^39^ However, DFO has an extremely short half‐life of approximately 20‐30 minutes in human plasma so that it must be infused continuously for 8‐24 h per day for several days each week, which hampers its potential applications due to its arduous regimen for patients and low compliance. Systemically administered DFO can hardly reach therapeutic concentration and target tumors at sufficient dosage, and local DFO can induce overexpression of HIF1α for tumor survival, which limits its use as an effective antitumor agent in clinical therapy.^40^ Lang et al have reported that the encapsulation of DFO within polymeric nanoparticles could successfully extend its circulation half‐life in a preclinical mouse model.^41^ To target the DFO‐induced HIF1α, DFO and HIF1α inhibitor lificiguat (also known as YC1) were codelivered. The YC1‐targeted DFO‐encapsulated nanoplatform significantly eliminated excess systemic iron and improved the antitumor efficacy in vivo (Figure 1).^41^

### 3.2 Nanotherapeutics harnessing Fenton reaction for ferroptosis induction

**FIGURE 2:**
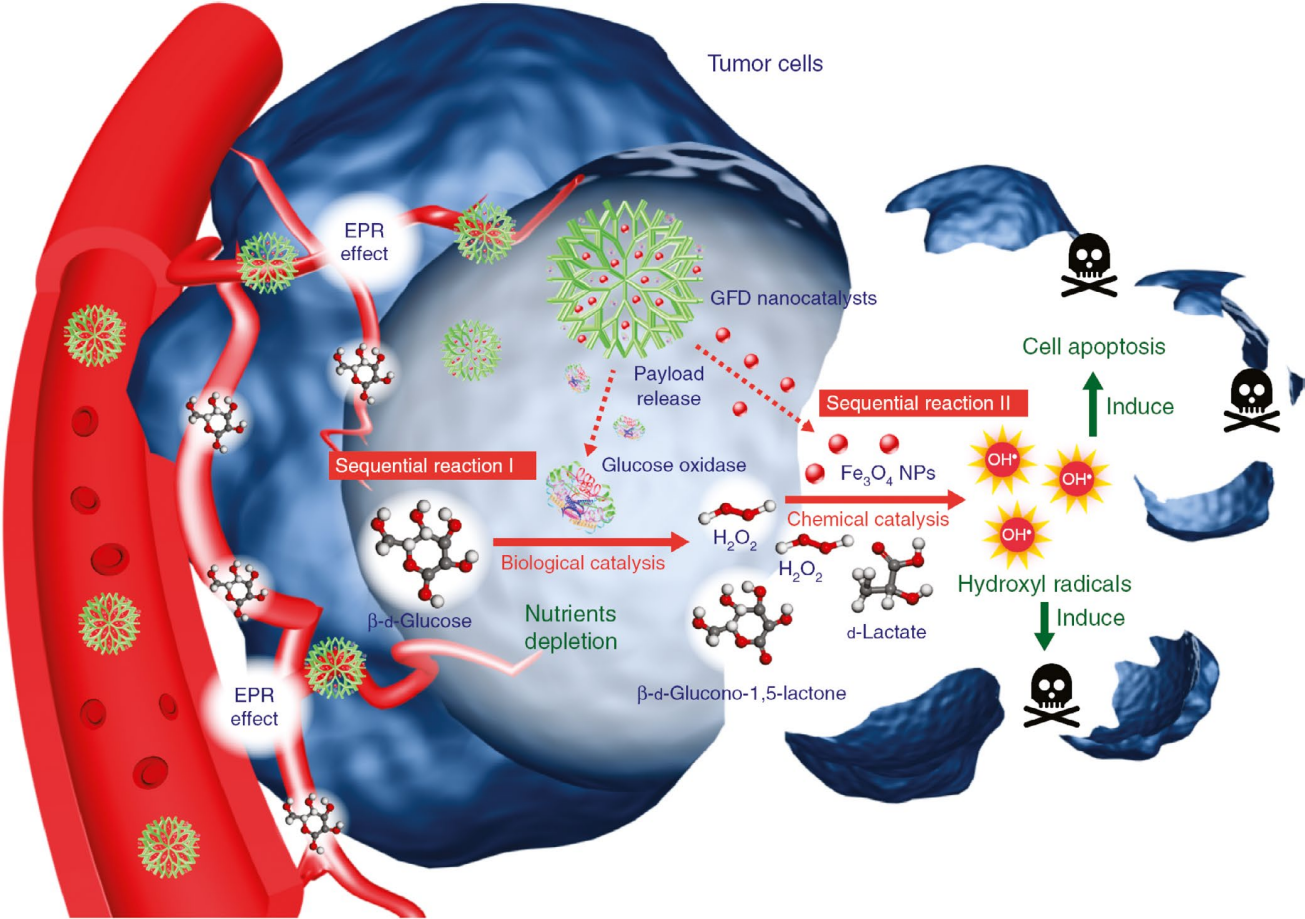
Schema of the sequential catalytic‐therapeutic mechanism of glucose oxidase (GOD)‐Fe_3_O_4_ integrated into dendritic mesoporous silica nanoparticle (GFD) nanocatalysts on the generation of hydroxyl radicals for cancer therapy. Initially, GFD nanocatalysts penetrate tumor tissues through the enhanced permeability and retention effect. The GOD in GFD nanocatalysts can effectively consume glucose through enzyme‐catalyzed biological reactions, which also produce abundant H_2_O_2_ molecules in situ. The generated H_2_O_2_ products are utilized as substrates and can be further catalyzed by the co‐encapsulated ultra‐small Fe_3_O_4_ nanoenzymes through Fenton‐like reactions, resulting in the production of highly toxic hydroxyl free radicals for cell apoptosis and death induction. Reprinted from Huo et al (2017),^55^ with permission from Springer Nature. NP, nanoparticle

Ferroptosis, discovered in 2012, is a type of programmed cell death different from apoptosis, autophagy, and necrosis. It is an iron‐dependent type of nonapoptotic cell death driven by cell metabolism and iron‐dependent lipid peroxidation^42^ and plays an important role in the pathogenesis and progression of cancer.^43^, ^44^, ^45^ Circulating Fe^3+^ is introduced into cells through TFR1 and then converted into Fe^2+^ in the endosome. Excessive Fe^2+^ will produce ROS and cause lipid peroxidation through the Fenton reaction, which will lead to ferroptosis.^46^ Iron oxide nanoparticles have been found to inhibit the proliferation of tumor cells by elevated iron‐mediated oxygen radicals, indicating the potential strategy to induce ferroptosis in the treatment of cancer.^47^ Ferumoxytol (Feraheme, AMAG Pharmaceuticals, Inc.) is approved by the FDA for the treatment of iron deficiency diseases and has achieved great success in cancer therapy, such as antileukemia therapy.^48^, ^49^ Ferumoxytol markedly inhibited the expression of ferroportin in vitro and in vivo and induced a potent antitumor effect on leukemias through regulating the intracellular iron content, promoting the differentiation of M1 macrophages, producing ROS and inflammatory factors, and then activating caspase‐3‐triggering cell apoptosis and ferroptosis in leukemic cells.^48^ Injection of ferumoxytol effectively inhibited the initiation of breast cancer and reduced the risk of liver and lung metastasis.^49^ In recent years, the development of iron‐based nanoparticles has advanced in cancer therapy through inducing Fenton reaction‐dependent ferroptosis, which provides a future perspective on this emerging field.^50^, ^51^, ^52^ However, due to the lack of H_2_O_2_, the limited acidity, and inadequate oxygen supply in the hypoxic tumors, it is urgent to develop an effective strategy to achieve the optimal conditions for the Fenton reaction for potent ferroptosis‐mediated cancer therapy.^53^ Fenton reaction‐based ROS‐manipulated nanocatalytic medicine has been developed and extensively studied in the treatment of cancer. The iron‐dependent lipid peroxide generator triggered the coupling sequence of glutathione and iron redox and then directly induced Fenton reaction‐dependent ferroptosis.^54^ Large‐pore and biodegradable dendritic silica nanoparticles encapsulated with natural glucose oxidase (enzyme catalyst) and ultra‐small Fe_3_O_4_ nanoparticles (inorganic nanoenzyme, Fenton reaction catalyst) can effectively consume glucose in the tumor cells and increase the concentration of H_2_O_2_ in the mildly acidic tumor microenvironment. These nanoparticles with sequential catalytic reactions could trigger apoptosis and cell death in tumor cells by producing highly toxic hydroxyl free radicals, which increased their antitumor capability (Figure 2).^55^ SnFe_2_O_4_ nanocrystals triggered the catalase‐mediated intracellular heterogeneous Fenton reaction and effectively inhibited the proliferation of colon cancer, but not in normal colon tissue, indicating the highly selective precision of nanomedicine in cancer therapy through Fenton reaction‐mediated ferroptosis.^56^ However, ferroptosis‐driven nanotherapeutics for cancer treatment might not meet the demands of individual patients under different circumstances. Combination with other therapeutic approaches, including chemotherapy, photothermal therapy, and immune regulation, could provide additional benefits in the clinic, which will be introduced in the following sections.

### 3.3 Ferroptosis‐inducing cancer nanomedicine combined with chemotherapy

The antitumor mechanism of chemotherapeutic drugs depends on apoptosis induction. Therefore, the combination of chemotherapy with ferroptosis, a different type of programmed cell death, will increase the therapeutic efficacy against cancer growth through synergistic killing effects on cancer cells. A nanolongan delivery system, one core (upconversion nanoparticles, UCNP) in one gel particle (in Fe^3+^), showed deep tumor penetration ability and efficiently reduced Fe^3+^ to Fe^2+^. Due to their valence state transition, the nano longan was destroyed in the tumor, which led to the rapid release of Fe^2+^ and doxorubicin (DOX) as a combination of ferroptosis induction and chemotherapy, thereby achieving excellent anticancer activity.^57^ In addition, calcium‐based biomimetic nanomaterials have been reported as potential nanotherapeutics by synergistically inducing tumor ferroptosis and apoptosis.^58^ A nanoassembly based on amorphous calcium carbonate (ACC) for tumor‐targeted ferroptosis therapy produced a synergistic effect and induced tumor cell death through complementary ferroptosis/apoptosis mechanisms where the fully degradable ACC substrate could interact with both DOX and Fe^2+^. Doxorubicin‐induced NADPH oxidase activated ROS and Fe^2+^ simultaneously for a synergistic effect to amplify ferroptosis damage. Dual ferroptosis/apoptosis treatment ultimately leads to effective tumor growth inhibition.^58^ Therefore, ferroptosis‐induced cancer nanomedicine combined with chemotherapy provides a novel therapeutic strategy and pharmaceutical platform for cancer therapy and showed significant and promising clinical prospects for treating cancer.

### 3.4 Ferroptosis‐induced cancer nanomedicine combined with photothermal therapy

**FIGURE 3:**
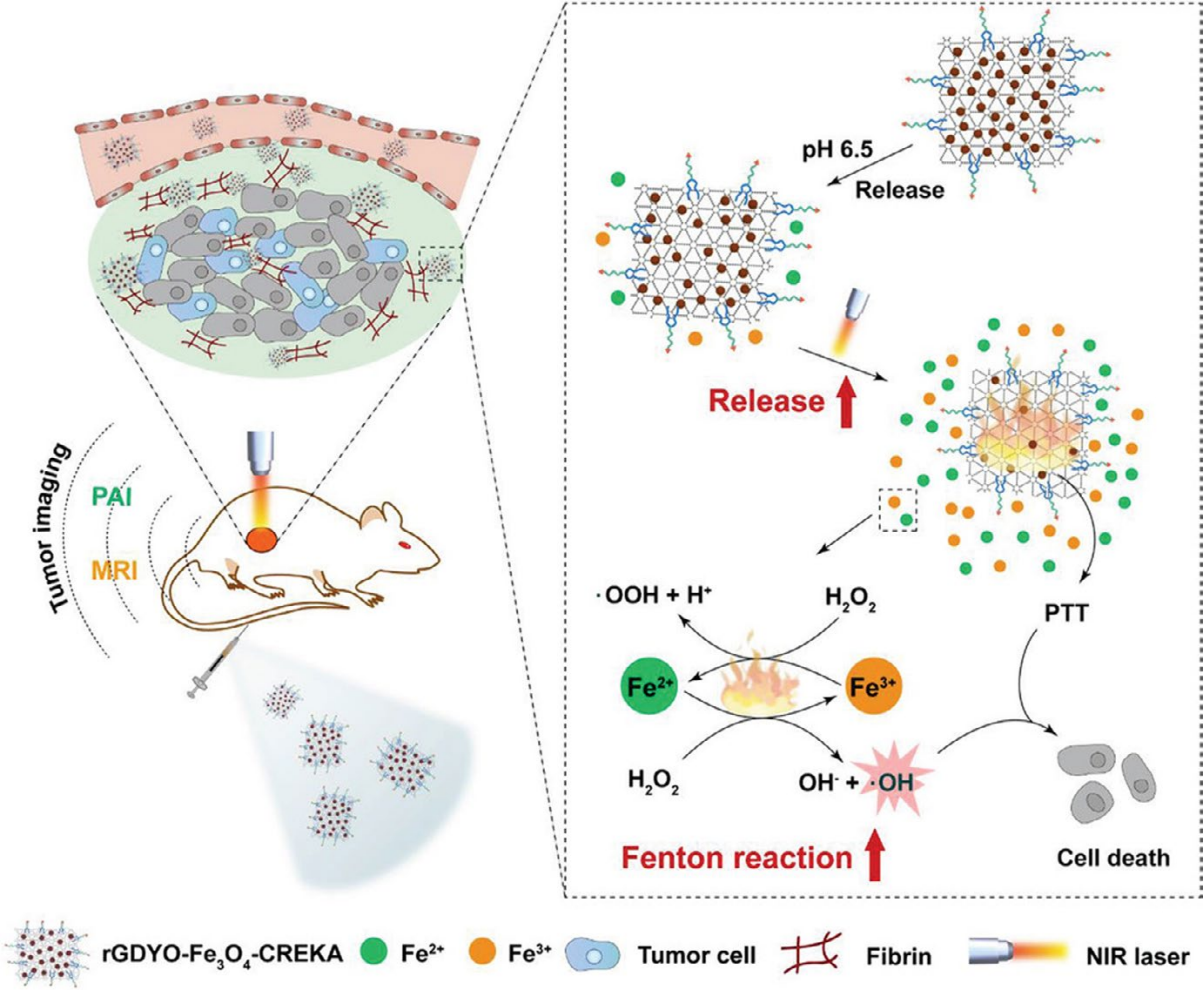
Schematic illustration of tumor‐targeted sponge iron (TTIS, rGDYO‐Fe3O4‐CREKA)‐mediated tumor therapy by a photothermally enhanced Fenton reaction. The TTIS can accumulate in tumor tissue by underdecorated tumor‐targeting polymer to enable tumor imaging with photoacoustic imaging and MRI. The accumulated TTIS can release Fe^2+^/Fe^3+^ ions in the acidic tumor environment. Additionally, TTIS can elevate the temperature of the tumor site upon near‐infrared irradiation to achieve effective photothermal therapy (PTT). Moreover, the heat produced during PTT can accelerate the release of Fe^2+^/Fe^3+^ ions and increase the production of ·OH through an enhanced Fenton reaction, thereby leading to a photothermally enhanced Fenton reaction‐mediated tumor therapy. Reprinted from Min et al (2020),^61^ with permission from Wiley

Near‐infrared (NIR) laser‐induced photothermal therapy (PTT) is used as a minimally invasive treatment technique by converting the optical energy of photothermal agents into thermal energy to kill tumor cells in an effective local tissue. Iron‐chelated semiconductor multicomposite nanoparticles have been reported in the treatment of cancer through photothermal “ferroptosis” under the guidance of photoacoustic imaging (PAI). This synergistic effect not only minimized the amount of iron but also effectively inhibited the growth of tumors in mice.^59^ A hybrid semiconductor nanoenzyme (HSN) showed high photothermal conversion efficiency for the second near‐infrared (NIR‐II) photothermal ferrotherapy, guided by PAI. Hybrid semiconductor nanoenzyme contains an amphiphilic semiconducting polymer as a light‐to‐heat converter, a photoacoustic emitter, and an iron‐chelated Fenton catalyst. Under light irradiation, the heat generated by HSN did not only induce cytotoxicity but also enhanced the Fenton response. Increased hydroxyl radical production promoted ferroptosis and cell apoptosis oxidized HSN and converted it into tiny fragments, thereby improving permeability within the tumor. This noninvasive seamless therapy can amplify the therapeutic effect, including deep ablation, reduced expression of metastasis‐related proteins, and inhibition of the metastasis of the primary tumor to distant organs. This hybrid polymer nanoenzyme strategy overcomes the limitations of ferrotherapy and PTT by NIR‐II PAI‐guided combined cancer therapy.^60^ Recently, tumor‐targeted sponge iron (TTIS) nanocomposite based on graphene oxide has been developed with a high affinity to iron for accumulation in tumor tissues, which enhanced the PTT and Fenton reaction‐mediated antitumor effect.^61^ First, TTIS modified with tumor‐targeting polymer significantly accumulated in tumor tissues and empowered the tumor photoacoustic and magnetic resonance imaging. Second, TTIS is an effective PTT agent along with excellent photothermal conversion efficiency. In addition, the PPT‐mediated photothermal energy accelerated the release of iron ions in TTIS and improved the efficiency of the Fenton reaction, leading to PTT and Fenton reaction‐mediated synergetic tumor therapy (Figure 3). Therefore, ferroptosis‐inducing cancer nanomedicine combined with photothermal therapy acts as a promising noninvasive therapeutic technique for cancer therapy.

### 3.5 Ferroptosis‐inducing cancer nanomedicine combined with immune regulation

Immunotherapy has revolutionized cancer treatment and rejuvenated the field of tumor immunology through restoring and fortifying the immune system to fight cancer. Tumor immunotherapy is playing an increasingly important role in the treatment of cancer. Results from clinically immunotherapeutic trials have been encouraging and seen in various malignant tumors through immune checkpoint inhibitors, therapeutic Abs, cytokines, cancer vaccines, immune cell therapy, and small molecule inhibitors.^62^ Recent studies in vitro and in vivo have shown that increased ferroptosis can make immunotherapy treatment more effective at killing cancer.^63^ The combination of tumor immunotherapy and ferroptosis‐based therapy has achieved an enhanced antitumor efficacy and showed significant clinical benefit in patients with cancer. Zhang et al^64^ constructed a biomimetic magnetosome through click chemistry to implement the synergism of ferroptosis and immunomodulation in cancer treatment. The magnetosome was composed of Fe_3_O_4_ magnetic nanoclusters as the core and a prefabricated white blood cell membrane as a cloak. The transforming growth factor‐β (TGF‐β) inhibitor was loaded inside the membrane, and the programmed cell death protein‐1 (PD‐1) Ab was used as an anchor on the membrane surface. After intravenous injection, membrane camouflage resulted in long‐term circulation. The superparamagnetic nanocluster core was guided by MRI for tumor targeting. Once into the tumor, the PD‐1 Ab and TGF‐β inhibitor worked together to form an immunogenic microenvironment, which increased the amount of H_2_O_2_. The Fenton reaction in polarized M1 macrophages promoted the reaction between Fenton and Fe ions released by nanoclusters. The generated hydroxyl free radicals (•OH) subsequently led to the invasion of tumor cells by lethal ferroptosis, and the exposed tumor antigens, in turn, improved the immunogenicity of the tumor. Therefore, the synergistic effect of immune regulation and ferroptosis in this cyclical manner leads to a strong therapeutic effect with minimal abnormalities, which supports this ferroptosis/immunological regulation synergy as a promising combination of anticancer therapy.^64^ In addition, Pang and Yang et al reported another biomimetic magnetic nanoplatform, Fe_3_O_4_‐sulfasalazine@platelet (Fe_3_O_4_‐SAS@PLT), which induced effective ferroptosis and enhanced the tumor immunogenicity for improved cancer immunotherapy. Fe_3_O_4_‐SAS@PLT was camouflaged by mesoporous magnetic nanoparticles, and platelet membranes loaded with sulfasalazine and inhibited the glutamate‐cysteine antiporter system. Fe_3_O_4_‐SAS@PLT‐mediated ferroptosis significantly improved the efficacy of programmed cell death and immune checkpoint blockade therapy, and repolarized macrophages from the immunosuppressive M2 phenotype to the antitumor M1 phenotype. Application of Fe_3_O_4_‐SAS@PLT in vivo achieved a durable tumor elimination effect in the mouse 4T1 metastatic tumor model, which is expected to have great potential in the clinical treatment of metastatic tumors.^65^ Biomimetic nanoparticles are considered the primary choice for the combination treatment of ferroptosis and immunotherapy. Studies have shown that the radiation‐induced bystander effect was mainly mediated by microparticles released by irradiated tumor cells, which enhanced tumor immunogenicity through ferroptosis and caused a broad range of antitumor effects.^66^ Oxidative stress induces the release of KRASG12D protein from pancreatic ductal adenocarcinoma (PDAC) cells and causes autophagy‐dependent ferroptosis. The autophagy‐dependent KRASG12D protein was released from PDAC cells in the form of exosomes, and induced fatty acid oxidation in a STAT3‐dependent manner, then drove and polarized macrophages into a tumor‐promoting phenotype similar to M2. Inhibiting the release of KRASG12D and the subsequent uptake of KRASG12D‐containing exosomes by immune cells can reduce the growth of PDAC tumors.^67^ An interesting common phenomenon in the above studies is that ferroptosis induced by nanoparticles was accompanied by the polarization of macrophages from M2 to M1. This is because the ROS produced by the Fenton reaction are essential for the induction and maintenance of M1 macrophage polarization. Reactive oxygen species could activate nuclear factor‐κB and p38 MAPK signaling pathways to promote the pro‐inflammatory cytokines for M1‐type macrophage polarization. This implies that ferroptosis could have some inevitable connection with macrophages, where lipid peroxidation induced by ferroptosis plays an essential role. Therefore, in the treatment of tumors, it is plausible to generate a synergistic therapeutic effect through the combination of ferroptosis and immune regulation.

## 4 CONCLUSIONS AND PERSPECTIVES

Although some iron metabolism‐targeted nanotherapeutics show promise in cancer treatment, more potent therapeutic strategies are desired and are increasingly exploited as attractive techniques based on iron metabolism regulation. For example, in this review, a promising approach is to combine iron‐targeted therapy with other therapeutics, such as combining ferroptosis‐inducing nanotherapeutics with chemotherapy, photothermal therapy, or immunomodulation. In light of the new generation of single‐chain Abs against TFR1 that effectively reduce the level of intracellular iron and markedly antagonize the development of leukemia,^68^ conjugation of such functional Abs with chemo‐ or cytotoxic drugs, such as DOX, cisplatin, ricin A chain, or diphtheria toxin, or conjugation of the Ab with micelles and dendrimers that carry antineoplastic drugs, could provide bispecific therapy against the tumor. With the advantage that magnetic nanomaterials contain an iron source per se, magnetic targeting probes, magnetocaloric, and MRI reagents combined with iron metabolism regulators could further enhance antitumor effects. A study has shown that combining high‐temperature chemotherapy using iron nanoparticles with magnetic guidance represented a powerful method for cancer treatment.^69^ In particular, biomimetic nanoparticles, due to their high biocompatibility and low toxicity, have received increasing attention in the field of cancer treatment, including iron‐targeting cancer therapy. Exosomes secreted by cancer‐associated fibroblasts have been found to be involved in the regulation of ferroptosis in cancer cells through microRNA‐522, which provides a novel idea for improving the sensitivity of gastric cancer chemotherapy.^70^ Functional ferritin nanoparticles that assembled by original or modified (ie, iron‐deleted) ferritin subunits could target TFR1 overexpressed in the tumor microenvironment while delivering chemodrugs or MRI agents for tumor therapy or imaging purposes.^71^

Although achievements have been made using nanoenabled iron therapy, the majority of iron‐regulatory nanoparticles remain in the preclinical stage. Currently, thermal ablation of prostate cancer using iron nanoparticles, called Magnablate (developed by University College London Hospitals), is being trialed in the clinic (NCT02033447, ClinicalTrials.gov), but it has not yet been approved by the FDA.^72^ Challenges are lining up to be overcome, such as improving the surface functionalization to enhance their active targeting, preventing drug leakage during circulation, optimizing the amount and speed of drug release in the lesion area, reducing the aggregation of metal nanoparticles, and reducing the adverse effects. Further in‐depth understanding of the molecular mechanisms of iron in cancer and advanced techniques in exploring the multifunctional nanomaterials aimed at iron metabolism regulation are demanded for successful tumor treatments.

## CONFLICT OF INTEREST

The authors have no conflict of interest to declare.

## ETHICAL APPROVAL

This work did not involve investigations on human subjects, nor experiments involving animals.
